# The Microbiological and Clinical Characteristics of Invasive *Salmonella* in Gallbladders from Cholecystectomy Patients in Kathmandu, Nepal

**DOI:** 10.1371/journal.pone.0047342

**Published:** 2012-10-15

**Authors:** Sabina Dongol, Corinne N. Thompson, Simon Clare, Tran Vu Thieu Nga, Pham Thanh Duy, Abhilasha Karkey, Amit Arjyal, Samir Koirala, Nely Shrestha Khatri, Pukar Maskey, Sanjay Poudel, Vijay Kumar Jaiswal, Sujan Vaidya, Gordon Dougan, Jeremy J. Farrar, Christiane Dolecek, Buddha Basnyat, Stephen Baker

**Affiliations:** 1 Oxford University Clinical Research Unit, Patan Academy of Health Sciences, Kathmandu, Nepal; 2 The Hospital for Tropical Diseases, Wellcome Trust Major Overseas Programme, Oxford University Clinical Research Unit, Ho Chi Minh City, Vietnam; 3 Centre for Tropical Medicine, Oxford University, Oxford, United Kingdom; 4 The Wellcome Trust Sanger Institute, Hinxton, Cambridge, United Kingdom; 5 Patan Hospital, Kathmandu, Nepal; Indian Institute of Science, India

## Abstract

Gallbladder carriage of invasive *Salmonella* is considered fundamental in sustaining typhoid fever transmission. Bile and tissue was obtained from 1,377 individuals undergoing cholecystectomy in Kathmandu to investigate the prevalence, characteristics and relevance of invasive *Salmonella* in the gallbladder in an endemic area. Twenty percent of bile samples contained a Gram-negative organism, with *Salmonella* Typhi and *Salmonella* Paratyphi A isolated from 24 and 22 individuals, respectively. Gallbladders that contained *Salmonella* were more likely to show evidence of acute inflammation with extensive neutrophil infiltrate than those without *Salmonella*, corresponding with higher neutrophil and lower lymphocyte counts in the blood of *Salmonella* positive individuals. Antimicrobial resistance in the invasive *Salmonella* isolates was limited, indicating that gallbladder colonization is unlikely to be driven by antimicrobial resistance. The overall role of invasive *Salmonella* carriage in the gallbladder is not understood; here we show that 3.5% of individuals undergoing cholecystectomy in this setting have a high concentration of antimicrobial sensitive, invasive *Salmonella* in their bile. We predict that such individuals will become increasingly important if current transmission mechanisms are disturbed; prospectively identifying these individuals is, therefore, paramount for rapid local and regional elimination.

## Introduction

Enteric fever is a systemic infection caused by the invasive bacteria *Salmonella* Typhi (*S.* Typhi) and *Salmonella* Paratyphi A (*S*. Paratyphi A). The disease is contracted by the ingestion of fecal material containing the pathogens [1]. The disease remains common in regions with poor standards of hygiene and sanitation, with global estimates suggesting that 27 million people are affected annually, of which 200,000 people die [2]. With adequate treatment >95% of patients recover completely from typhoid [1]. However, an estimated 2–5% of individuals infected with *S.* Typhi develop a sustained infection of the gallbladder [3]. These individuals are referred to as ‘carriers’, and like the infamous ‘typhoid Mary’ [4], are outwardly asymptomatic, continue to intermittently shed organisms for a prolonged period and often have no recollection of an acute episodes of typhoid [1].

During acute typhoid, invasive *Salmonella* cross the intestinal epithelial barrier, invade and survive within macrophages, eventually reaching the bone marrow, liver, pancreas and spleen [5]. Invasion of the gallbladder occurs either directly from the blood or by retrograde spread from the bile [1]. In a subset of individuals infected with *S.* Typhi, the organisms chronically colonize the gallbladder and carriers shed these organisms intermittently into the intestinal lumen and thus in the feces. It is gallbladder colonization and fecal shedding that form a central dogma for the transmission and persistence of typhoid fever. As a consequence of the internal localization of organisms, this dogma is difficult to challenge in humans and the host-restricted nature of the relevant pathogens make carriage difficult to replicate precisely in non-mutant mouse models [6]. As a result, data regarding the prevalence, bacteriology and mechanisms of carriage are sparse. The only population-based study estimating chronic *Salmonella* carriage in an endemic setting is from Chile where investigators gathered data from autopsies, calculating a carriage rate of 694 per 100,000 [3].

Investigations of *Salmonella* carriage suggest that the propensity to become a chronic carrier follow the typical epidemiology of gallbladder disease. Thus, the likelihood of carriage increases with age and is more common in females [7]. Existing data also imply that individuals with gallstones or other gallbladder abnormalities are at increased risk of carriage [8]. These epidemiological theories are supported by laboratory-based investigations, which have shown that *Salmonella* can form biofilms and survive for prolonged period on gallstones [9], [10].

There remains a significant burden of typhoid fever across Asia, yet the understanding of *Salmonella* carriage in these populations is limited. We have found previously that *S*. Paratyphi A can be isolated from the gallbladders of patients undergoing cholecystectomy and we suggested that carriage of invasive *Salmonella* play a pivotal role in the persistence of these pathogens in Kathmandu, Nepal [11]. We aimed to define the microbiology and epidemiology of invasive *Salmonella* carriage in Kathmandu. We demonstrate that *S*. Typhi and *S*. Paratyphi A are present in the gallbladder in a high concentration, are less common than other Gram-negative organisms, are not associated with lymphocytic infiltration in the gallbladder tissue, and do not exhibit resistance to multiple antimicrobials.

## Results

### Microbiological Examination of Bile from Cholecystectomy Patients

From June 2007 until October 2010, a total of 1,496 patients underwent cholecystectomy for acute or chronic cholecystitis at Patan hospital in Kathmandu. From the 1,496 patients, bile samples from 1,377 individuals were obtained and subjected to microbiological examination; 119 (8%) patients either denied consent or were unavailable for recruitment. A Gram-negative organism was isolated from 20% (274/1,377) of the bile samples. *E. coli*, *Salmonella spp.* and *Klebsiella spp.* were the most commonly isolated organisms, found in 78 (5.7%), 48 (3.5%) and 41 (3.0%) of the bile samples, respectively (Table 1). The remainder of the culture positive bile samples contained a range of organisms including *Psuedomonas spp*., *Acinetobacter spp*., *Enterobacter spp., Citrobacter freundii, Vibrio spp.* and *Serratia marcescens* (Table 1). Of the 48 *Salmonellae* isolated, 24 (1.7%) were *S*. Typhi, 22 (1.6%) were *S*. Paratyphi A and two (0.1%) were *S. enterica* group C.

**Table 1:** Antimicrobial resistance patterns of Gram-negative organisms from the bile of patients undergoing cholecystectomy.

Organism	Patients	Antimicrobial resistance n (%)								
	n (%)	Amoxicillin	Cefotaxime	Ciprofloxacin	Ofloxacin	Chloramphenicol	Cotrimoxazole	Gentamycin	Amikacin	Nalidixic Acid
Typhi	24 (1.7)	0/23 (0)	0/24 (0)	0/24 (0)	0/24 (0)	0/22 (0)	4/23 (17.4)	0/11 (0)	0/0 (0)	11/22 (50.0)
Paratyphi A	22 (1.6)	0/22 (0)	0/22 (0)	1/22 (4.5)	1/22 (4.5)	2/22 (9.1)	0/22 (0)	0/16 (0)	0/1 (0)	16/19 (84.2)
Other	2 (0.1)	1/2 (50.0)	0/2 (0)	0/2 (0)	0/2 (0)	1/2 (50)	0/2 (0)	0/1 (0)	0/0 (0)	1/2 (50.0)
**Total ** ***Salmonella***	**48 (3.5)**	**1/47 (2.1)**	**0/48 (0)**	**1/48 (2.1)**	**1/48 (2.1)**	**3/46 (6.5)**	**4/47 (8.5)**	**0/28 (0)**	**0/1 (0)**	**28/43 (65.1)**
*Escherichia coli*	78 (5.7)	32/76 (42.1)	12/76 (15.8)	23/77 (29.9)	21/77 (27.3)	14/69 (20.3)	22/76 (28.9)	4/40 (10.0)	0/39 (0)	20/42 (47.6)
*Klebsiella spp*	41 (3.0)	36/39 (92.3)	9/39 (23.1)	6/39 (15.4)	6/38 (15.8)	12/38 (31.6)	11/39 (28.2)	4/24 (16.7)	1/25 (4.0)	9/18 (50.0)
*Pseudomonas spp*	33 (2.4)	23/31 (74.2)	4/31 (12.9)	4/31 (12.9)	4/31 (12.9)	21/28 (75.0)	21/30 (70.0)	2/20 (10.0)	1/18 (5.6)	9/13 (69.2)
*Acinetobacter spp*	19 (1.4)	14/19 (73.7)	7/19 (36.8)	6/19 (31.6)	4/19 (21.1)	11/19 (57.9)	9/19 (47.4)	5/9 (55.6)	4/10 (40.0)	6/11 (54.5)
*Enterobacter spp*	21 (1.5)	16/19 (84.2)	6/20 (30.0)	2/20 (10.0)	1/20 (5.0)	3/19 (15.8)	3/20 (15.0)	1/8 (12.5)	0/5 (0)	2/14 (14.3)
Other	34 (2.5)	14/18 (77.8)	5/20 (25.0)	3/20 (15.0)	2/17 (11.8)	5/18 (27.8)	3/18 (16.7)	1/4 (25.0)	1/3 (33.3)	5/19 (26.3)
**Total Non-** ***Salmonella***	**226 (16.4)**	**135/202 (66.8)**	**43/205 (21.0)**	**44/206 (21.4)**	**38/202 (18.9)**	**66/191 (34.6)**	**69/202 (34.2)**	**17/105 (16.2)**	**7/100 (7.0)**	**51/117 (43.6)**

Forty-six *Salmonella* isolates were available for antimicrobial susceptibility testing by disc diffusion. Fifty-nine percent (27/46) of the *Salmonella* isolates were resistant to nalidixic acid, and a single *S*. Paratyphi A isolate was resistant to both nalidixic acid and ciprofloxacin. All *S*. Typhi and *S*. Paratyphi A strains were susceptible to ceftriaxone, chloramphenicol, gatifloxacin and ofloxacin and we identified no multi-drug resistant (MDR) (resistant to chloramphenicol, ampicillin and co-trimoxazole) isolates. One *S. enterica* group C isolate demonstrated resistance to nalidixic acid, ceftriaxone, gatifloxacin and chloramphenicol (Table 1).

Baseline data, stratified by microbiological culture result are shown in Table 2. Notably, fitting with the typical epidemiological characteristics of cholelithiasis, 77% (1,066/1,377) of the patients were female and the median age was 39 years (range: 16 to 76 years). The median age of those with *Salmonella* in their bile was 35 years (range: 18 to 67 years) and 73% were female. It is noteworthy that none of the pre-surgical stool cultures from any patients were *Salmonella* positive and, when questioned, only 15% (7/48) of the *Salmonella* bile-positive patients had a memorable history of typhoid, none of which had been confirmed by microbiological culture. From available records, 16% (7/43) of *Salmonella* bile-positive patients reported >5 days of fever on entry, 7% (3/46) were admitted with jaundice, 5% (2/41) had a palpable gallbladder and 4% (2/45) were admitted with pancreatitis.

**Table 2:** The baseline characteristics of the *Salmonella* positive, culture negative and the culture positive for non-*Salmonella* bile culture groups.

		Women		Men		Typhoid fever history		Surgery	
Culture group	Patients	Age	Patients	Age	Patients	Clinical diagnosis	Culture diagnosis	Elective	Acute
	n (%)	Median (range)	n (%)	Median (range)	n (%)	n (%)	n (%)	n (%)	n (%)
*Salmonella* positive	48 (3.5)	34.5 (20–67)	35 (72.9)	38 (18–57)	13 (27.1)	7 (14.6)	0 (0)	33 (68.8)	10 (20.8)
Culture positive for non-*Salmonella*	226 (16.4)	39 (16–76)	167 (73.9)	46 (11–80)	50 (22.1)	35 (15.5)	12 (5.3)	200 (88.5)	10 (4.4)
Culture negative	1103 (80.1)	38 (16–76)	864 (78.3)	44 (11–75)	214 (19.4)	176 (16.0)	29 (2.6)	983 (89.1)	61 (5.5)
Total	1377 (100)	39 (16–76)	1066 (77.4)	45 (11–80)	277 (20.1)	218 (15.8)	41 (3.0)	1216 (88.3)	81 (5.9)

### Bacterial Load of Salmonella in Bile

To quantify the bacterial load in the bile, real-time PCR was performed on total nucleic extracted from the bile of six *S.* Paratyphi A positive individuals and 12 *S*. Typhi positive individuals. All qualitative serovar specific PCR data corresponded precisely with the culture data. The median target copy numbers/bacterial loads were 9.3×10^4^ (IQR 5×10^4^–2.3×10^5^) CFU/ml^−1^ for *S*. Paratyphi A and 5.2×10^4^ (IQR 2×10^4^–7.28×10^5^) CFU/ml^−1^ for *S*. Typhi. The difference in bacterial load between the two organisms was non-significant (*p = *0.93; Mann-Whitney U test), yet, were approximately two and three orders of magnitude greater than those previously reported in bone marrow and blood, respectively [12], [13].

### Hematological and Biochemical Characteristics

The 1,377 individuals undergoing cholecystectomy were divided into three groups on the basis of their bile culture results: *Salmonella* positive, culture negative, and culture positive for non-*Salmonella.* Individuals that were *Salmonella* positive were more likely to have experienced continuous right upper-quadrant pain (10%, 5/48) compared to those that were culture negative (3%, 37/1,151) (*p = *0.008, chi squared test) and those that were culture positive for non-*Salmonella* (2%, 5/214) (*p = *0.008, chi squared test). Hematology and biochemistry data from the patients were compared using the Mann-Whitney U test (Table 3). There was no significant difference in liver enzyme or bilirubin levels between the *Salmonella* positive group and the other two groups. Yet, the *Salmonella* positive group had a higher median neutrophil count and a lower median lymphocyte count than the culture negative group and the culture positive non-*Salmonella* group (Table 3).

**Table 3:** The haematological and the biochemical characteristics of the *Salmonella* positive, culture negative and the culture positive for non-*Salmonella* bile culture groups.

Hematology	Culture negative			Culture positive non-*Salmonella*			*Salmonella* positive			p1*	p2*
	n	median	IQR	n	median	IQR	n	median	IQR		
Total cell (×10^3^/µL)	953	7.9	6.6–9.5	188	7.85	6.45–10.15	42	9.45	6.4–14	**0.025**	0.058
Neutrophil (×10^3^/µL)	917	66	58–74	177	65	58–75	41	72	60–82	**0.012**	**0.042**
Lymphocyte (×10^3^/µL)	914	31	24–38	175	31	23–38	40	24.5	17–36	**0.007**	**0.040**
Monocyte (×10^3^/µL)	270	1	1–2	70	1.5	1–2	9	1	1–2	0.676	0.848
Eosinophil (×10^3^/µL)	641	2	1–4	131	2	1–4	22	2	2–4	0.999	0.515
Basophil (×10^3^/µL)	54	0	0–1	14	0	0–0	1	6	–	0.058	**0.020**
Total bilirubin (mg/mL)	965	0.8	0.68–1	190	0.8	0.7–1	42	0.86	0.7–1.1	0.119	0.280
Conjugated bilirubin (mg/mL)	950	0.2	0.19–0.26	186	0.2	0.18–0.24	41	0.2	0.2–0.28	0.414	0.419
AST (u/L)	961	30	23–41	190	29.5	23–40	42	28	24–38.9	0.878	0.986
ALT (u/L)	960	30	21.9–43	188	29.5	21–43.5	42	30.5	24–41	0.898	0.953
ALP (u/L)	941	122	82–209	188	150.5	94.5–232.5	42	124.5	96–191	0.622	0.276
Amylase (u/L)	136	61.5	41.5–252.5	20	57.5	38–86.5	9	87	34–190	0.867	0.437

* Mann-Whitney U test, boldface indicates p≤0.05.

p1: Comparing culture-negative to *Salmonella*-positive patients.

p2: Comparing culture positive for non-*Salmonella* to *Salmonella*-positive patients.

IQR: Interquartile range.

### Surgical and Histopathological Characteristics

The major surgical and post-surgical characteristics of the gallbladders from the three groups were compared using Fisher’s exact test (Table 4). The majority of *Salmonella* positive individuals had gallstones (96%, 46/48); yet, there was no significant difference in the proportion of individuals with gallstones between the three groups. We did, however, identify several gallbladder characteristics that were associated the presence of *Salmonella*. Namely, gallbladder distension and inflammation was more frequently observed in the *Salmonella* positive group than the culture negative group and the non-*Salmonella* culture positive group (Table 3). Furthermore, the presence of an empyema (pus within the gallbladder cavity) was also more common in the *Salmonella* positive group than the other two groups. Inflammation was more likely to be due to polymorphonuclear infiltration than lymphocytic infiltration in the *Salmonella* infected gallbladder tissue, with 13% (6/48) of the *Salmonella* positive gallbladder specimens having massive neutrophil infiltrate near the lumen, compared to 4% (51/1,151) and 5% (10/214) of the culture negatives and the non-*Salmonella* culture positives, respectively. Furthermore, an additional 15% (7/48) of the *Salmonella* positive gallbladder specimens had acute-on-chronic cholecystitis (neutrophil infiltrate near the lumen with lymphocyte infiltrate and dysplasia in the mucosa) compared to 5% (10/214) and 7% (14/214) of the culture negatives and the non-*Salmonella* culture positives, respectively (Table 3.). Correspondingly, chronic inflammation without large neutrophil infiltrate was not observed in gallbladder tissue from the *Salmonella* positive group.

**Table 4:** The gallbladder characteristics within the *Salmonella* positive, culture negative and the culture positive for non-*Salmonella* bile culture groups.

Characteristic	Culture negative	Culture positive non-*Salmonella*	*Salmonella* positive	*p1* *	*p2* *
	n = 1,103	n = 214	n = 48		
**Gallbladder tissue thickness**					
Thick (>4 mm)	173 (15.7)	36 (16.8)	10 (20.8)	0.350	0.602
Normal (4 mm)	493 (44.7)	86 (40.0)	21 (43.8)		
Thin (<4 mm)	74 (6.7)	12 (5.6)	1 (2.1)		
**Gallbladder size**					
Contracted	108 (9.8)	25 (11.7)	1 (2.1)	**0.026**	**0.035**
Distended	221 (20.0)	52 (24.3)	15 (31.3)		
**Gall stones**					
None	19 (1.7)	6 (2.8)	3 (6.3)	0.101	0.481
Single	344 (31.2)	62 (29.0)	14 (29.2)		
Multiple	684 (62.0)	133 (62.1)	28 (58.3)		
**Pathology**					
Inflammation	93 (8.4)	17 (7.9)	8 (16.7)	**0.046**	0.060
Empyema	90 (8.2)	21 (9.8)	10 (20.8)	**0.003**	**0.033**
Sludge	57 (5.2)	8 (3.7)	1 (2.1)	0.338	0.581
Mucocele	50 (4.5)	7 (3.3)	1 (2.1)	0.427	0.664

* Fisher’s exact test, boldface indicates p≤0.05.

p1: Comparing culture-negative to *Salmonella*-positive patients.

p2: Comparing culture-positive, *Salmonella*-negative to *Salmonella*-positive patients.

## Discussion

The mechanism of gallbladder infection/colonization remains contentious, and it is unknown if *Salmonella* promote gallbladder damage during chronic infection or if the organisms exploit existing gallbladder damage to stimulate colonization [14]. Bile is typically sterile, and consists of organic and inorganic compounds, bile acids, cholesterol, phospholipids and the pigment biliverdin. Sterility is partially maintained by the secretion of IgA and mucus, preventing bacterial survival and adhesion to the surface of the lumen and the major bile duct, respectively [15]. Here, we found a wide array of organisms in the bile of individuals undergoing cholecystectomy, some of which have been previously isolated from the gallbladder [16]–[18]. Again, whether these organisms functionally stimulate cholecystitis or cholelithiasis, or merely have the ability to colonize damaged gallbladders, remains unclear. Our data confirm that non-*Salmonellae* organisms, with a spectrum of pathogenic potential, are as equally adept at colonizing the gallbladder and surviving within the bile as typhoidal *Salmonella*. Yet, non-*Salmonellae* appear not to stimulate the same pathology as *Salmonella*; *Salmonella* infected tissue was more commonly associated with systemic and local acute inflammatory responses. Mouse experiments, utilizing *Salmonella* Typhimurium, have shown that *Salmonella* can replicate within the epithelial cells of the gallbladder [19], and that colonized gallbladders displayed evidence of the epithelial destruction and local neutrophil infiltrate. Here, we also find extensive neutrophil infiltrate, yet are unable to confirm if the bacteria are damaging the tissue or colonizing previously damaged tissue. However, as shown by an increased prevalence of gallbladder distention, right upper quadrant pain, empyema and a raised systemic neutrophil count, there is an evident association of invasive *Salmonella* in the gallbladder with an acute inflammatory response.

We have previously noted the presence of individuals in Kathmandu with *Salmonella* in their gallbladder, highlighting the presence of *S*. Paratyphi A [11]. The role of chronic carriage of *S.* Paratyphi A has received much less attention than that of *S*. Typhi and it is unknown as to what extent chronic gallbladder carriage is contributing to the increasing burden of *S.* Paratyphi A across many parts of Asia [20]. Enteric fever caused by *S*. Paratyphi A increased from 17.5% (155/885) in 1993 to 34% (926/2,718) in 2003 in the location of this study [21].

Furthermore, we found an almost equal ratio of *S*. Typhi and *S*. Paratyphi A (1∶0.9) isolated from bile, yet the isolates from blood from acutely infected patients over the same period is lower (1∶0.4) [22]. This disparity may result from a multitude of factors, but may predict that *S*. Paratyphi A is more adept at inducing carriage in this population, or, once in the gallbladder, may be more likely to induce an acute inflammatory response, requiring a surgical intervention, than S. Typhi.

We found that 3.5% of the individuals undergoing gallbladder surgery had invasive *Salmonella* in their bile in this area with a high incidence of enteric fever [23]. A report from a similar patient demographic in India suggest an equivalent rate of <5%, and in Chile, 7.3% of bile cultures were found to be positive for *Salmonella* [*7*]. The long-term carriage of invasive *Salmonella* in the gallbladder is thought to be central to the maintenance and transmission of these human-restricted pathogens [14]. However, data from our previous work in Kathmandu suggests that direct transmission plays a negligible role in acute infections, and we have hypothesized that carriers merely act as a reservoir for maintaining local strain diversity in areas of high endemicity [22]. Here, we found antimicrobial resistance to only nalidixic acid in the *Salmonella* from the gallbladder. Although nalidixic acid resistance often precedes resistance to other fluoroquinolones, these isolates were susceptible to gatifloxacin and ofloxacin. Firstly, these data show that infection with an antimicrobial resistant organism is not likely to be associated with *Salmonella* carriage. Secondly, if one considers nalidixic acid resistance as a proxy marker of contemporary strains, the organisms in the gallbladder have probably been there for some time (i.e. from a period when nalidixic acid resistance was less prevalent) [21], [24]. Nalidixic acid resistance is a growing problem in Kathmandu. From an ongoing clinical trial enrolling enteric fever patients over the last two years at Patan Hospital, 80% (171/214) of invasive *Salmonella* isolates demonstrated resistance to nalidixic acid, which is greater than the proportion (59%) found from bile isolates in the current study (unpublished data). This evidence supports our current hypothesis of gallbladder carriage playing a limited role in the acute transmission of typhoid in Kathmandu.

Whilst we argue that in locations such as Kathmandu, the role of carriers in typhoid fever transmission may be negligible, it is reasonable to suggest that those shedding invasive *Salmonella* play a vital important role in low transmission setting. In the USA, up to 30% of typhoid fever infections are anticipated to result from contact with a chronic carrier [25]. Therefore, these individuals will become increasingly important as indirect transmission this area begins to subside after the introduction of an effective intervention strategy. However, currently there is no appropriate diagnostic test for the detection of long-term carriers [26]. Bile cultures from string devices are considered effective [27], but are impractical for screening large cohorts [28]. The presence of gallbladder disease is, perhaps, currently the best clinical predictor of carriage of invasive *Salmonella* [*7*]. However, it remains unclear as to why some patients progress to become chronic shedders and others do not. The development of a rapid diagnostic for the detection of invasive *Salmonella* carriage should accelerate regional elimination of typhoid and add insight into the epidemiological role of these individuals.

One of the major caveats of our study that limits the generalizability of our findings is the fact that our passively acquired patient population may not accurately reflect the general population of Kathmandu. Additionally, all patients in the study had some form of gallbladder abnormality, although it is unclear whether such abnormalities had been induced by the infecting organisms. Nevertheless, in the absence of an alternative methodology, our study represents a reasonable estimation of the burden and mechanism of invasive *Salmonella* carriage.

In conclusion, we have calculated a prevalence of 3.5% of invasive *Salmonella* in bile from patients undergoing cholecystectomy in Kathmandu, Nepal. We demonstrate that *S*. Paratyphi A is almost as prevalent as *S.* Typhi in the gallbladder in this population and that carriage is not driven by antimicrobial resistance. The overall role of invasive *Salmonella* carriage in settings such as Kathmandu is not understood, and we suggest that organisms in the gallbladder may not play a dominant role in acute typhoid fever in this location. We predict, however, that carriers will become more important if current transmission mechanisms are disturbed; prospectively identifying these individuals is paramount for rapid local and regional elimination.

## Methods

### Ethics Statement

This study was conducted according to the principles expressed in the Declaration of Helsinki and was approved by the institutional ethical review boards of Patan Hospital, The Nepal Health Research Council and The Oxford University Tropical Research Ethics Committee (OXTREC, Reference number: 2108). All enrollees were required to provide written informed consent for the collection and storage of all samples and subsequent data analysis. In the case of those under 18 years of age, a parent or guardian was asked to provide written informed consent.

### Setting and Study Population

The study was conducted at Patan Hospital, a 318-bed government hospital located in the Lalitpur Sub-Metropolitan City in the Kathmandu valley, Nepal. Patan Hospital provides both emergency and elective inpatient services. Typhoid fever is a common complaint at Patan Hospital and *S*. Typhi and *S*. Paratyphi A are the most common bacteria cultured from blood of febrile patients in this location. Antimicrobials are available without prescription in the community in a variety of public and private outlets and there are numerous private physician clinics where patients may seek advice and clinical diagnosis for febrile disease. There has been no widespread implementation of a typhoid vaccine in this area, yet a generic typhoid Vi vaccine is available for purchase in some health care settings. However, at the time of this investigation there was limited community uptake of the vaccine.

The surgical department performs approximately 400 cholecystectomies annually. For the purposes of this study, consecutive patients admitted to the surgical ward from June 2007 to October 2010 for either open cholecystectomy or laparotomy surgery for symptomatic cholelithiasis between 8 am and 4 pm were approached for participation. All patients who gave written informed consent were eligible for the study; there were no exclusion criteria. All enrollees were also required to provide written informed consent for the collection, use and storage of the tissue removed during surgery samples. A questionnaire related to the patient’s health and demographics was administered prior to surgery along with a stool sample for microbiological culture. Surgeons collected bile samples and gallbladder tissue during the procedure.

### Gallbladder Morphology and Histopathology

Patients were routinely examined by ultrasonography before surgery to assess the presence of gallstones and to detect inflammation. The surgeon performing the procedure curated a report, assessing the thickness of the gallbladder wall (stratified into three categories, thick: >4 mm, normal: 4 mm and thin: <4 mm), the presence and the number of gallstones, the presence and characteristics of fluid (pus: empyema, mucoid/clear/watery: mucocele and sludge) and overall morphology (contracted or distended). Hematocrit, total leukocytes with differential count, total bilirubin, conjugated bilirubin, alanine aminotransferase (ALT), aspartate aminotransferase (AST), and amylase were measured prior to surgical intervention. All extracted tissue was subjected to a histopathological examination to assess/confirm the extent of the inflammation; all histopathology was performed by the same skilled technician who was blinded to presence or absence of bacteria within the bile. All sections we examined by light microscopy after staining with heamatoxylin and eosin. Inflammation was identified by tissue morphology and the presence of neutrophils (acute) and lymphocytes (chronic).

### Microbiological Culture, Antimicrobial Susceptibility Testing and Real-time PCR

Bile and stool were collected for culture from all patients undergoing cholecystectomy. Bile was inoculated into equal volumes of Selenite F broth and Peptone broth and incubated at 37°C overnight. Broths were sub-cultured onto MacConkey agar and Xylene Lysine Deoxycholate (XLD) agar. After overnight incubation at 37°C the plates were examined for the growth of Gram-negative bacteria and colonies were identified by standard microbiological methods and identified by API20E manufactured by bioMerieux, Inc. *S*. Typhi and *S*. Paratyphi A isolates were confirmed by slide agglutination by specific antisera (Murex Biotech, Biotech, England). The antimicrobial sensitivity profile was performed by Kirby Bauer disc diffusion method using standard BSAC and CLSI guidelines [29]. The antimicrobials tested were amoxicillin, chloramphenicol, co-trimoxazole, nalidixic acid, ciprofloxacin, ofloxacin, ceftriaxone and gatifloxacin. The minimum inhibitory concentrations (MICs) were performed for nalidixic acid, ciprofloxacin, ofloxacin and azithromycin by E-test (AB Biodisk, Sweden). Susceptibility to ciprofloxacin and ofloxacin were evaluated using newly suggested susceptibly breakpoints for these antimicrobials; ≥0.125 µg/ml and ≥0.25 µg/ml for ciprofloxacin and ofloxacin respectively [30]. Real-time PCR was performed using a standard curve for quantitation as previously described [12], using DNA extracted from 200µl bile samples as template.

### Data Analysis

Data were entered into a database using Excel 2007 (Microsoft) and analyzed using Stata/IC version 9.2 (StataCorp, TX, USA). Chi-square and Fisher’s exact tests were used to compare proportions between groups and Mann-Whitney U tests were used for continuous non-parametric data. P-values ≤0.05 were considered to be statistically significant.
